# Identification of Capacitive MEMS Accelerometer Structure Parameters for Human Body Dynamics Measurements

**DOI:** 10.3390/s130911184

**Published:** 2013-08-22

**Authors:** Vincas Benevicius, Vytautas Ostasevicius, Rimvydas Gaidys

**Affiliations:** Institute for Hi-Tech Development, Faculty of Mechanical Engineering and Mechatronics, Kaunas University of Technology, Studentu 65-209, Kaunas LT-51369, Lithuania; E-Mails: vincas.benevicius@gmail.com (V.B.); rimvydas.gaidys@ktu.lt (R.G.)

**Keywords:** capacitive, accelerometer, MEMS, optimization, proof-mass, L-shaped beam

## Abstract

Due to their small size, low weight, low cost and low energy consumption, MEMS accelerometers have achieved great commercial success in recent decades. The aim of this research work is to identify a MEMS accelerometer structure for human body dynamics measurements. Photogrammetry was used in order to measure possible maximum accelerations of human body parts and the bandwidth of the digital acceleration signal. As the primary structure the capacitive accelerometer configuration is chosen in such a way that sensing part measures on all three axes as it is 3D accelerometer and sensitivity on each axis is equal. Hill climbing optimization was used to find the structure parameters. Proof-mass displacements were simulated for all the acceleration range that was given by the optimization problem constraints. The final model was constructed in Comsol Multiphysics. Eigenfrequencies were calculated and model's response was found, when vibration stand displacement data was fed into the model as the base excitation law. Model output comparison with experimental data was conducted for all excitation frequencies used during the experiments.

## 1. Introduction

A new technology usually begins with experimentation. Anything that is ever built must be designed first. This is immediately followed by modeling as one wants to know how well the device works before it is built so that expensive experimentation can be reduced. Modeling techniques and tools enable analysis of an existing design. The design itself is largely dependent on the experience, expertise and the creativity of the designer. Optimal synthesis techniques have the potential to reduce this reliance on the human designer by automatically generating designs matching user-specified requirements [1]. Microaccelerometer synthesis algoritms have been successfully applied to the automatic layout of surface-micromachined accelerometers [2]. A prerequisite for synthesis was a set of lumped-parameters models that adequately linked device behaviour with physical design variables. Optimal synthesis enabled exploration of the entire design space given specific user-specified constraints, as has been shown with the accelerometer example. Concepts of multidisciplinary design and optimization were introduced in [3] for overall and preliminary design of a microgyroscope. Optimization of the design of such a system requires a thorough understanding of the coupling effects of their working environments, their physical structural parameters, their electronic construction, and their fabrication processes. A tuning fork gyroscope was taken as an exemple to demonstrate the principle for optimizing the necessary multidisciplinary design procedures in designing MEMS. Tay *et al.* presented a single crystal silicon low-g open loop microaccelerometer designed and fabricated through a spreadsheet optimization methodology [4]. The paper begins with the theoretical formulation and analysis of the differential capacitive accelerometer. The effects of the electrostatic spring constant on the natural frequency and sensitivity of the accelerometer have been thoroughly discussed. The ratiometric error for this system has been optimized. A topology optimization-based approach is used in [5] for the design of micromachined force amplifiers in inertial sensor applications. Technological constraints require that the results of the topology optimization mechanisms should be converted to beam element models and subjected to a further size and shape optimization. The dependence of geometrical and mechanical parameters on the optimization is studied. Paper [6] deals with the possibility of vibration mode control of MEMS devices having large potential in various micro-sensor/actuator applications. The presented numerical analysis focuses on the first three flexural vibration modes and their influence on dynamic characteristics. Advantages and drawbacks deriving from the use of MEMS accelerometers for hand-arm and whole-body vibration measurements are evaluated in [7]. Metrological performances of different transducers are assessed through the identification of their frequency response function, linearity, floor noise and sensitivity to thermal and electromagnetic disturbances. Experimental results highlighted a standard instrumental uncertainty with the single frequency calibration procedure. The temperature effect was negligible and the electromagnetic disturbances sensitivity was comparable to that of the piezoelectric accelerometers. The footstep vibration signals are measured in [8] by floor-mounted MEMS accelerometers deployed tangent to wall sides, for estimating the level of indoor physical activity. With growing concern about obesity in older adults and disabled people, this paper deals primarily with the estimation of energy expenditure in the human body. It also supports the localization of footstep sources, extraction of statistical parameters on daily living patterns, and identification of pathological gait patterns. Unlike other sensors such as cameras or microphones, MEMS accelerometer sensors can measure many biomedical signatures without invoking personal privacy concerns. Accelerometers in [9] are commonly used in motion analysis systems to enable researchers to conduct studies outside of the traditional laboratory environment; however the available systems tend to be bulky and unsuitable for long-term studies. Therefore, a need exists for a physically robust, yet compact motion analysis system that can be easily worn for an extended time period without disrupting the person's normal range of motion. The aim of this research work is to investigate the applicability of optimization techniques for identification of MEMS accelerometer structures for human body measurements, when any information available from the accelerometers' producer is limited. The paper is organized as follows: Selection of accelerometer structure is made in Section 2, accelerometer model and its validation are presented in Section 3. Concluding remarks are provided in the final Section 4.

## 2. Selection of Accelerometer Structure

The availability of a system capable of automatically classifying the physical activity performed by a human subject is extremely attractive for many applications in the field of healthcare monitoring and in developing advanced human-machine interfaces. The information on the human physical activity is valuable in the long-term assessment of biomechanical parameters and physiological variables. Serious estimation errors may occur when wearable sensor systems composed of motion sensors, such as accelerometers, are used without any regard to what the subject is actually doing [10].

**Figure 1.**
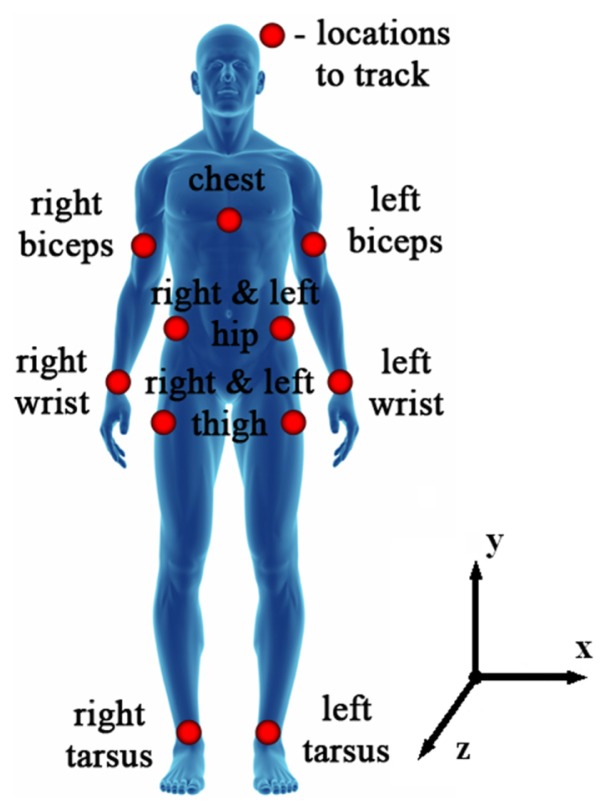
Set of locations to be tracked on the human body during experiments.

In order to define digital acceleration signal characteristics and guidelines for the hardware selection and safe filtering thresholds to be used, body acceleration signals analysis must be performed in order to identify possible maximum accelerations and signal bandwidth. Such an analysis can only be performed with quantitative analysis methods such as photogrammetry, which allows precise and reliable measurements from images [11]. Cameras and markers are suggested for this analysis as the technique with least 3D space restrictions compared to radiography or magnetic resonance imaging. An experiment when the subject starts walking very slowly and gradually increases his speed to the level he can still cope with would completely cover the range of acceleration signals that can be produced during everyday physical activities considering acceleration signal bandwidth and amplitude. The results of human body acceleration signal analysis could suggest some guidelines for the accelerometer to be chosen. Vertical movement is a major component in everyday physical activities. Walking, running, sitting up and down among them are most common movements. Body locations, as defined in Figure 1, were tracked using six ProReflex MCU 500 Type 170 241 cameras with Qualisys Track Manager Software from Qualisys and utilizing Treadmill “Vision Fitness Premier” model T9450 HRT. The ProReflex MCU uses a 680 × 500 pixel CCD image sensor. The use of CCD technology results in very low-noise data compared to a higher resolution CMOS sensor which has a considerably higher pixel noise levels. By using a patented sub-pixel interpolation algorithm, the effective resolution of the ProReflex MCU is 20,000 × 15,000 subpixels in a normal setup, in some cases enabling the ProReflex MCU to discern motions as small as 50 μm [12]. Experiments started with slow 0.8 km/h walking, was gradually increased to 13 km/h intensive running and decreased back to 0.8 km/h. Speed was changed in increments/decrements of 0.1 km/h with a delay of 5 s. Three cycles were performed for each test subject. All motion data (direction x, y and z) was captured with data sampling rate of 500 Hz to keep raw data at the maximum resolution that was allowed by the hardware. All captured data was filtered using 20 Hz low pass filter with 80 dB attenuation at 25 Hz as bandwidth of 20 Hz is where natural human movements occur.

Residual analysis was performed for every data set in the given bandwidth of 20 Hz by employing the MATLAB routine that was developed. During residual analysis error *e_f_* was calculated for every frequency between 1 Hz and 20 Hz:

(1) $$e_{f}=\max\left(\left|x_{i}-y_{i}\right|\right)$$

where *x_i_* is measured data sample, *y_i_* is filtered data sample using low pass filter with passband frequency *f*.

Values of *e_f_* were monitored while *f* was gradually decreased from 20 Hz in steps of 1 Hz. Safe passband frequency is considered such value of *f* that is captured as soon as *e_f_* starts to nonlinearly increase while *f* decrease. The analysis suggested safe pass band frequency of 16 Hz (Table 1).

The differentiator filter was applied twice to obtain accelerations for every marker that was tracked. Maximum accelerations observed during experiments are given in Table 2. Ledoux and Hillstrom during their research [13] obtained that peak-to-peak accelerations in the tarsus area (talus) were up to 6.75 g. This is similar to the measurements that were aqcuired during this research, however the scope of analyzed movement was not only walking but also running up to 13 km/h. Also the choise of data filtering might be the reason of different maximum values obtained as high frequencies are removed (or left).

According to Qu *et al.* [14] typical body acceleration amplitude can range up to 12 g. As industrial accelerometers that are available through major electronic components dealers (Farnell, Digikey, *etc.*) have ranges ±2 g, ±4 g, ±6 g, ±8 g, ±16 g, due to limitations in availability of ±12 g accelerometers, ±16 g acceleration data should be chosen.

**Figure 2.**
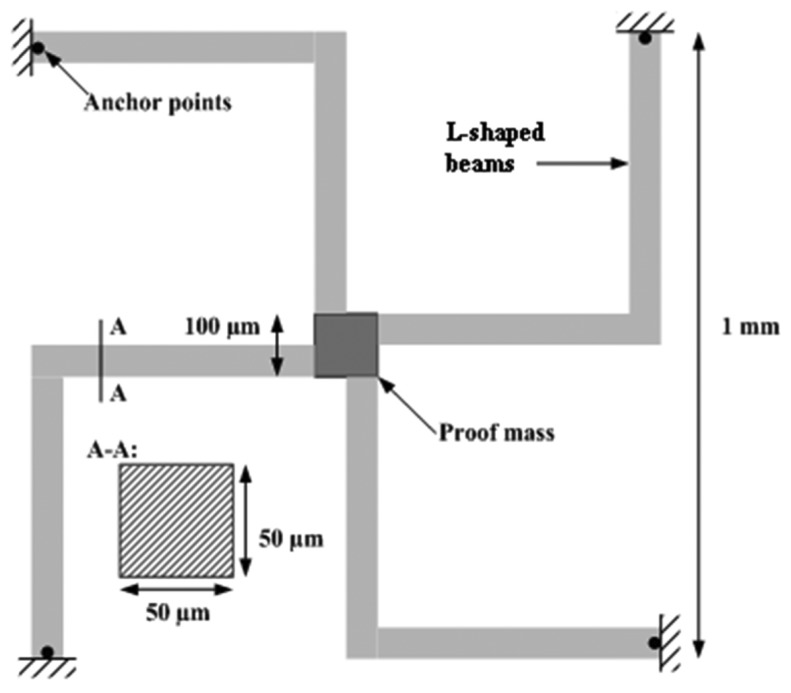
Computational scheme of MEMS accelerometer.

It is common for the MEMS accelerometer to have its sensing part fit into area of ∼1 mm^2^[10]. Thus the first model requirement is that sensing element must fit into area of 1–1.3 mm^2^. It is also common to use silicon as the primary material in the manufacturing process [15]. The manufacturing process also defines that the sensing part must be developed as a 2D structure. However, operation requirements specify that the sensing part should measure on all three axes as it is a 3D accelerometer and sensitivity on each axis must be equal, that's why the lumped models for the main components of the proof-mass support were not be chosen. According to the values of the observed acceleration magnitudes and resulting guidelines the accelerometer that is to be identified should have a measuring range of ±16 g. The total non-linearity from the sensing element, electronics and from other sources should not be more than 1% of full scale output (FSO). The accelerometer should have a bandwidth (±3 dB) of >100 Hz and the cross-axis sensitivity should be limited to a maximum of 1% of FSO. The accelerometers bias stability and hysteresis values are specified as 0.15% of FSO each. Finally the accelerometer should have a response time of less than 1 ms and it should perform over a temperature range of −20 to 80 °C. The configuration of the capacitive accelerometer should be chosen in such a way, that the proof-mass should be free from squeeze damping effects and supported in vacuum on all four sides by shaped beams, that permit for a proof-mass piston-like movement and remain parallel to electrodes at all accelerations as well. Also any geometrical change in the beam length due to temperature variation limits the proof-mass to in-plane rotation only and it does not experience any out of plane bending. This configuration reduces the overall accelerometer chip size thereby improving the per wafer yield and also reduces the non-linearity associated with support structures. The most favorable beam configurations for this purpose are L-shaped cantilever beams (Figure 2). According to Yoshida *et al*. such a beam tends to give better sensitivity [16]. Thus we define additional model requirement: sensing part model will be L-shaped beams as the primary structure with internal damping capability. Based on all collected requirements we define initial accelerometer model geometry that is given in Figure 2 and Table 3.

## 3. Accelerometer Model and Its Validation

To investigate the applicability of the optimization technique for identification of MEMS accelerometer structure parameters, the accelerometer (Figure 2) 3D finite element model was created using Comsol with Matlab finite element analysis software. MEMS operation requirements specify that sensing part should measure on all three directions as it is 3D accelerometer and sensitivity on each axis must be equal. Consequently the beam cross section and mass density of proof-mass material were chosen as structure parameters.

Requirement of model's sensitivity equality can be defined as follows:

(2) $$\left\{ \begin{array}{l} u\left(1,A\right)=u(2,A)=u(3,A)\\ u(i,A)\neq 0 \end{array}\right.$$

where *u*(*i*,*A*) is a displacement along axis *I* = 1,2,3 (defines axis x, y and z accordingly) when acceleration of magnitude *A* is applied to the model along the same axis.

Error function can be defined as:

(3) e(i,t)=|u¨(i,t)m−a(i,t)|

Here *I* = 1,2,3 and defines axis x, y and z accordingly; *ü_m_*(*i*, *t*) — acceleration that is showed by the model at the moment *t*. Here it is assumed that dynamic excitation is governed by *a*(*i*,*t*) = sin(2*πft*), where *f* is excitation frequency.

Optimization problem that is to be solved in respect of accelerometer beam cross section height and mass density of proof-mass material:

(4) $$\underset{t\geq 0}{\min}\left|{\ddot{u}}_{m}(i,t,h,\rho )-\frac{d^{2}(\sin(2\pi ft)}{dt^{2}}\right|$$

where *ü_m_*(*i*,*t,h,ρ*) —acceleration of proof-mass at the moment *t* with accelerometer beam cross section height *h* and mass density of proof-mass material *ρ*; *f*—excitation frequency.

Defined optimization problem has following constraints:

(5) $$\{\begin{array}{l} f\in (0;20]\\ u(i,A,h,\rho )=u(j,A,h,\rho ),i\neq j\\ u(i,A,h,\rho )\neq 0\\ A\in (-160;0)\cup (0;160)\\ i,j=1,2,3\\ h\in (0;10^{-4})\\ \rho \in (0;10^{4}) \end{array}$$

These constraints come both from requirements described earlier as well as real world conditions:

- *f* ∈ (0,20] : frequency is limited for example to 20 Hz;
- *u*(*i*, *A*, *h*, *ρ*) = *u*(*j*, *A*, *h*, *ρ*), *i* ≠ *j* : is the axis sensitivity requirement;
- *u*(*i*, *A*, *h*, *ρ*) ≠ 0 : a constraint to avoid the zero displacement condition where formally axis equal sensitivity requirement would be met;
- *A* ∈ (–160;0) ∪ (0;160) : maximum acceleration levels are limited to 160 m/s^2^ in both directions on any axis because it's common for industrial accelerometers to have measurement range of ±16 g;
- *h* ∈ (0;10^−4^) : beam cross section height constraint that corresponds to real world manufacturing process;
- *ρ* ∈ (0;10^4^) : mass density of proof-mass material constraint.

The width *w* of cross section of accelerometers beam is explicitly set to be 4 μm as such dimension is used for mechanical springs in the accelerometers design [15]. Taking into consideration the origin of the problem it is possible to solve the problem for only one value of *A*. All other values would give the same result because the requirement for equality in sensitivity across all axes is set throughout all accelerometers operation range. Thus *A* is set to 10 m/s^2^.

The problem in Equation (4) is redefined as:

(6) $$\underset{t\geq 0}{\min}\left|{\ddot{u}}_{m}(i,t,h,\rho )-\frac{d^{2}(\sin(2\pi ft)}{dt^{2}}\right|$$

(7) $$\begin{array}{l} K(h)u+C(h)\dot{u}=-M(h,\rho )\ddot{u}\\ u(0)=\dot{u}(0)=0\\ F_{z}(t)=F_{y}(t)=F_{z}(t)=Am_{a}\sin(2\pi ft) \end{array}$$

The constraints become:

(8) $$\{\begin{array}{l} f\in (0;20]\\ u(i,h,\rho )=u(j,h,\rho ),i\neq j\\ u(i,h,\rho )\neq 0\\ i,j=1,2,3\\ h\in (0;10^{-4})\\ \rho \in (0;10^{4}) \end{array}$$

Equation (7) is the governing equation of motion where *K, C, M*—matrices of structure stiffness, damping and mass, *u*,*u̇*,*ü* —vectors of displacement, velocity and acceleration. Body loads *F_z_*(*t*),*F_y_*(*t*),*F_z_*(*t*) prescribed as external force/volume in all direction with acceleration *A* multiplied by a mass *m_a_* of MEMS accelerometer. The hill climbing optimization technique, which belongs to the local search family, was chosen to solve the problem. Initial variable values for the optimization problem were *h* = 1 × 10^−5^ m, *ρ* = 2,000 kg/m^3^. Error function minimum (Equations 4 and 5) was reached as sensitivity equality was satisfied for *h* = 8.25·μm and mass density of proof-mass material of *ρ* = 9,083.2 kg/m^3^ also yielding the minimal error function value of 5.06 × 10^−10^ m. The material with nearest value of mass density is copper (Cu), with *ρ* = 8,960 kg/m^3^, therefore copper was chosen as proof-mass material. Final model properties are given below in Table 4.

Material model is isotropic. Silicon (Si) has Young's modulus of 170 GPa, Poisson ration of 0.28 and density of 2,329 kg/m^3^. Copper (Cu) has Young's modulus of 120 GPa, Poisson ratio of 0.34 and density of 8,960 kg/m^3^.

**Figure 3.**
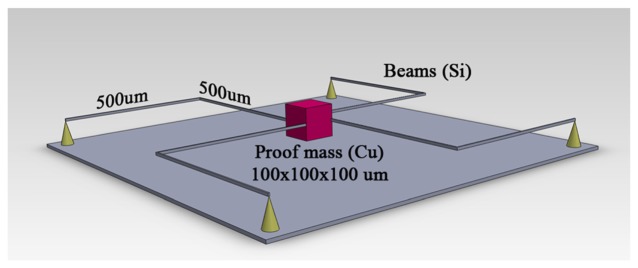
Final accelerometer model geometry.

**Figure 4.**
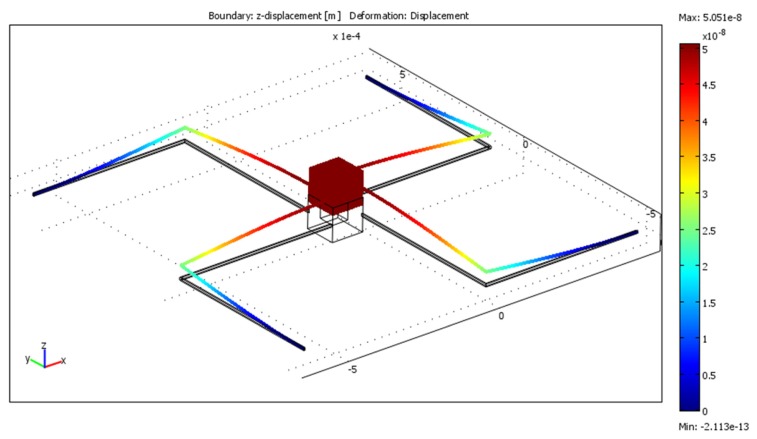
Displacements field in the z direction when 10 m/s^2^ acceleration is applied along the z axis.

**Figure 5.**
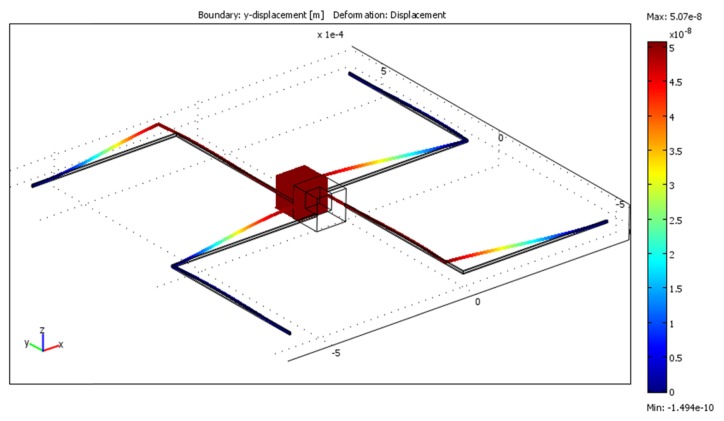
Displacement field in y direction when 10 m/s^2^ acceleration is applied along the y axis.

**Figure 6.**
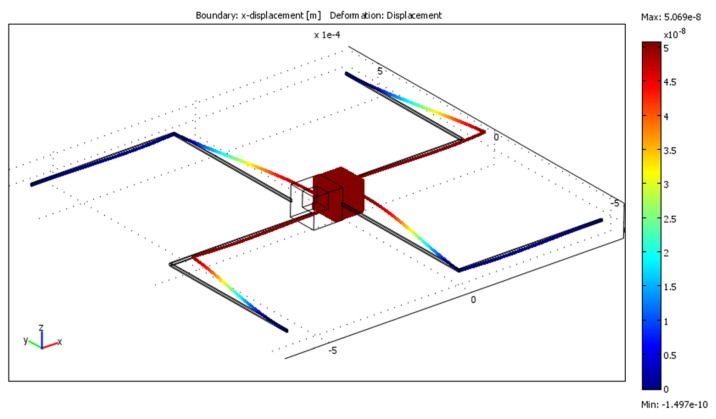
Displacement field in x direction when 10 m/s^2^ acceleration is applied along the x axis.

The final model (Figure 3) was constructed in Comsol Multiphysics following the geometry specifications that are given in Table 4. In order to verify that first resonant frequencies on every axis are outside the 20 Hz frequency range first three eigenfrequencies were computed. Results are given in Table 5. The response when acceleration of 10 m/s^2^ was applied to the model the displacement in z direction was 50.49 nm (Figure 4), displacement in y direction was 50.55 nm (Figure 5) and displacement in x direction was 50.54 nm (Figure 6).

Displacement in the x direction differs by 0.090% from the displacement in the z direction and by 0.006% from the displacement in the y direction while displacement in the y direction differs by 0.092%. These results clearly show that equal sensitivity on all axes is achieved in the model.

Experimental data that was acquired by monitoring uniaxial vibration stand movement was used to validate the accelerometer model. Vibration stand displacement data was fed into the model as the base excitation law and model's response was observed. Accelerations that were acquired from the model output were compared with vibration stand accelerations during experiment to see how well the model fits real world process.

Proof-mass displacements were analyzed for all acceleration ranges given by the optimization problem constraints (4). Results show that dependence between displacement and acceleration is linear, it means that acceleration conversion into digital signal is possible by measuring the capacitance between two plates where one is on the bounding box and other is on the proof mass. Electrical capacity between two plates is inversely related to the distance between plates and linearly depends on the area of overlap between these plates. Model output comparison with experimental data was conducted for all excitation frequencies that were used during experiment. Table 6 shows the results.

The given results show that the model fits the experimental data quite well. Although relative errors go a little over 9% of the acceleration amplitude, absolute errors does not exceed 0.12 m/s^2^. This shows that the model is valid and stable throughout the bandwidth of 20 Hz.

## 4. Conclusions

An accelerometer model was identified and validated. Silicon was used for L-shaped beams, copper for proof-mass. Overall top size was 1.23 mm^2^. L-shaped beam cross section size was 4 × 8.25 μm, proof mass size was 100 × 100 × 100 μm. It was shown that the model has first resonant frequencies (over 2,200 Hz) far from the bandwidth of interest (20 Hz). The model achieves equal sensitivity in all directions. Displacement in the x direction differed by 0.090% from displacement in the z direction and by 0.006% from displacement in the y direction when an acceleration of 10 m/s^2^ was applied to the accelerometer. Displacement in the y direction differed by 0.092% from displacement in the z direction. Validation of the model showed that in a frequency range up to 20 Hz modeled accelerations were up to 10% different from measured values with the absolute error being less than 0.12 m/s^2^.

## Figures and Tables

**Table 1.** Residual analysis results for walking/running task data samples.

**Data set**	**Suggested pass band frequency, Hz**
Chest	10
Back	10
Right biceps	11
Left biceps	11
Left hip	11
Right hip	10
Left wrist	10
Right wrist	10
Left thigh	16
Right thigh	16
Left tarsus	16
Right tarsus	16

**Table 2.** Maximum accelerations observed in three directions.

**Position**	**Max (|a_x_|), m/s^2^**	**Max (|a_y_|), m/s^2^**	**Max (|a_z_|), m/s^2^**
Chest	15.879	38.277	35.708
Back	22.437	38.587	18.750
Biceps	23.812	19.476	39.375
Hip	28.145	37.636	45.645
Wrist	31.656	32.347	71.700
Thigh	35.736	36.232	39.749
Tarsus	66.873	24.016	56.383

**Table 3.** Initial accelerometer model properties.

**Property**	**Value**
Material	Si
Overall size (top)	1 mm^2^
L-shaped beam cross section size	50 × 50 μm
Proof mass size	100 × 100 μm

**Table 4.** Final accelerometer model properties.

**Property**	**Value**
L-shaped beam material	Si
Proof mass material	Cu
Overall size (top)	1.23 mm^2^
L-shaped beam cross section size	4 × 8.25 μm
Proof mass size	100 × 100 × 100 μm

**Table 5.** Accelerometer model eigenfrequencies.

**First three eigenfrequencies**	**Value, Hz**
In x direction	2,238.81
In y direction	2,239.11
In z direction	2,244.52

**Table 6.** Accelerometer's model output comparison with vibration stand accelerations.

**Frequency, Hz**	**Excitation amplitude, mm**	**Acceleration amplitude, m/s^2^**	**Model acceleration amplitude, m/s^2^**	**Error, %**	**Error, |m/s^2^|**
1	0.8813	0.3480	0.3169	9.82%	0.0311
4	0.9517	0.6772	0.6296	7.56%	0.0476
7	0.9510	1.8397	1.7461	5.36%	0.0936
10	1.0822	4.2725	4.1853	2.08%	0.0872
14	1.0335	7.9967	7.8770	1.52%	0.1197
17	0.9891	11.2849	11.1665	1.06%	0.1184
20	0.9241	14.5920	14.4876	0.72%	0.1044
